# CEPsh glia development is not required for general AWC identity or AWC asymmetry

**DOI:** 10.17912/micropub.biology.000636

**Published:** 2022-09-15

**Authors:** Rui Xiong, Yi-Wen Hsieh, Chiou-Fen Chuang

**Affiliations:** 1 Department of Biological Sciences, University of Illinois at Chicago, IL, USA; 2 Graduate Program in Neuroscience, University of Illinois at Chicago, IL, USA

## Abstract

The *Caenorhabditis elegans* VAB-3/Pax6 homeodomain protein was previously shown to play a role in both the development of cephalic sheath (CEPsh) glia and asymmetric differentiation of AWC olfactory neuron subtypes AWC ^ON^ /AWC ^OFF^ . Here we show that *vab-3* is not required for the specification of general AWC identity. We also show that some *vab-3* mutant alleles with defective CEPsh glia development displayed wild-type AWC asymmetry. These results suggest that *vab-3* has separable roles in CEPsh glia development and AWC asymmetry. Together, our results suggest that general AWC identity and AWC asymmetry are not dependent on the development of CEPsh glia.

**Figure 1. vab-3 has separable roles in CEPsh glia and AWC development:**
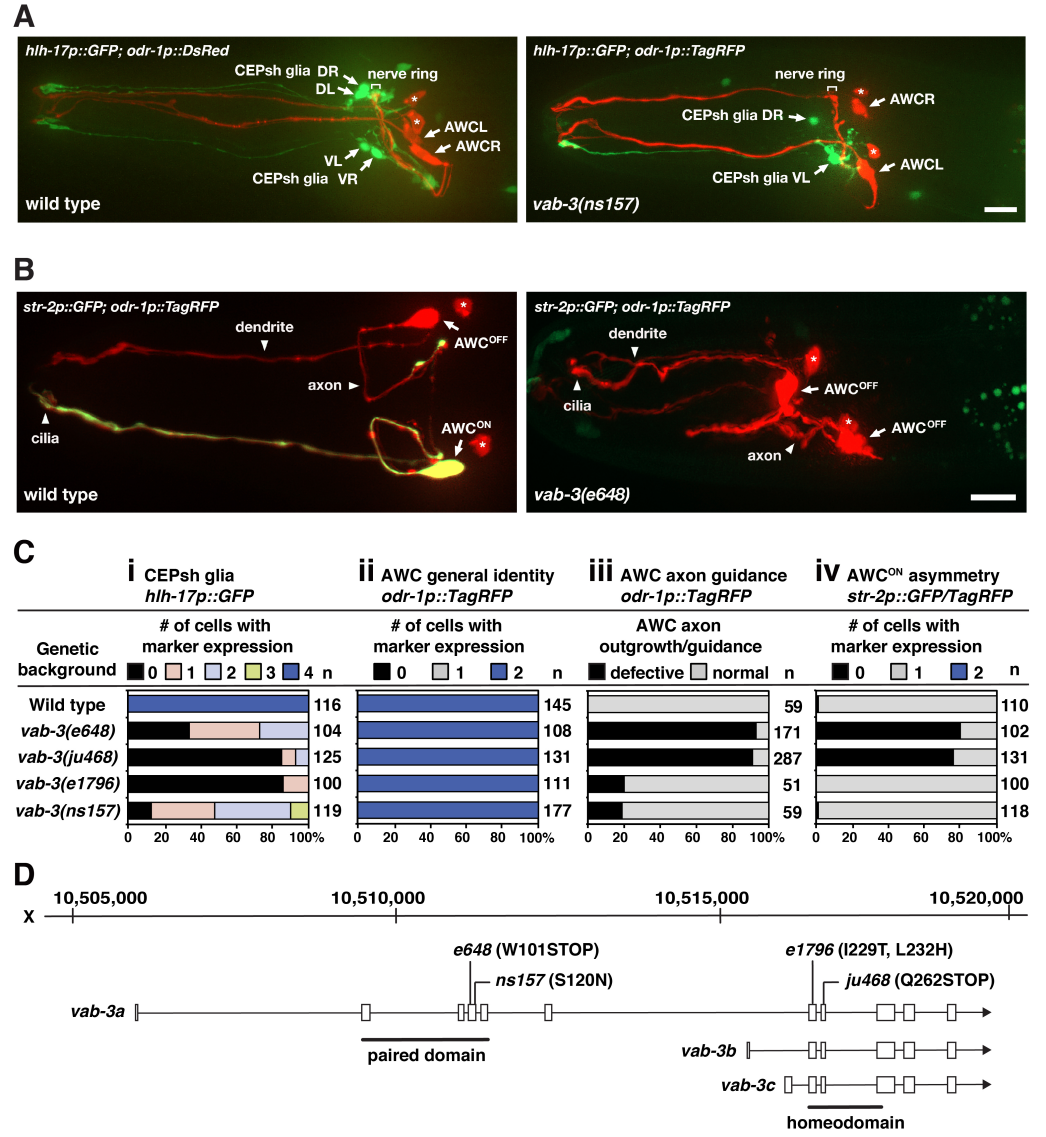
( **A** ) Representative images of wild type and *vab-3(ns157)* mutants expressing integrated transgenes of the CEPsh identity marker *hlh-17p::GFP* and general AWC identity marker *odr-1p::DsRed* or *odr-1p::TagRFP* in the adult stage. Arrows indicate the cell body of AWC and CEPsh glia; asterisks indicate the AWB cell body. Scale bar, 10 μm. Anterior to the left and ventral to the bottom. ( **B** ) Representative images of wild type and *vab-3(e648)* mutants expressing integrated transgenes of the AWC ^ON^ marker *str-2p::GFP* and general AWC identity marker *odr-1p::TagRFP* in the adult stage. Arrows indicate the cell body of AWC; asterisks indicate the AWB cell body. Scale bar, 10 μm. Anterior to the left and ventral to the bottom. ( **C** ) Expression of integrated transgenes of the CEPsh identity marker *hlh-17p::GFP* (i), general AWC identity and AWC axon outgrowth/morphology/guidance marker *odr-1p::TagRFP* (ii, iii), and the AWC ^ON^ asymmetry marker *str-2p::GFP* or *str-2p::TagRFP* (iv) in wild type and *vab-3* mutants. Animals were scored in adults. n, total number of animals scored. ( **D** ) Genomic structure and position of the *vab-3* locus in chromosome X. *vab-3* has three alternatively spliced isoforms, *vab-3a-c* (wormbase.org, release WS248).

## Description

The *C. elegans* nerve ring, considered to be the brain of the worm, is enveloped by sheet-like processes of four CEPsh glia (Figure 1A) (White et al., 1986). CEPsh glia was shown to control axon guidance and branching of sensory neurons, including AWC olfactory neurons, in the nerve ring (Yoshimura et al., 2008). It was also shown that normal axon guidance and AWC axon contact are required for asymmetric differentiation of the AWC neuron pair (Figure 1B) (Troemel et al., 1999). These findings prompted us to determine whether the development of CEPsh glia regulates two subsequent steps of AWC development, general AWC identity specification and AWC asymmetry. To address these questions, we studied candidate genes previously implicated in both CEPsh glia development and AWC development. *mls-2* , encoding an HMX/NKX MLS-2 transcription factor, and *vab-3* , encoding a homolog of homeodomain protein Pax6, have been shown to regulate the development of CEPsh glia (Figure 1A and 1C) (Yoshimura et al., 2008). We and others previously showed that *mls-2* plays a role in general AWC identity and AWC asymmetry (Kim et al., 2010; Hsieh et al., 2021). Since AWC asymmetry defects observed in *mls-2(lf)* mutants result from indirect defects in general AWC identity and direct defects in AWC asymmetry, it would not be straightforward to determine whether CEPsh glia development regulates AWC asymmetry using *mls-2* mutants. *vab-3* , like *mls-2* , is also required for AWC asymmetry (Figure 1B and 1C) (Troemel et al., 1999). But it was unknown whether *vab-3* regulates general AWC identity.

In addition to CEPsh glia development and AWC asymmetry, a wide array of *vab-3* mutations have been isolated to implicate its roles in other different aspects of development, including neuronal fate specification, anterior epidermal fate specification, head morphogenesis, axon guidance in the anterior nervous system, gonadal distal cell migration, blast cell fate specification in the rectal epidermis, and male tail morphogenesis (Lewis and Hodgkin, 1977; Hodgkin, 1983; Chamberlin and Sternberg, 1995; Chisholm and Horvitz, 1995; Zhang and Emmons, 1995; Nishiwaki, 1999; Troemel et al., 1999; Zallen et al., 1999; Cinar and Chisholm, 2004; Yoshimura et al., 2008; Doitsidou et al., 2010; Brandt et al., 2019). Here, four *vab-3* alleles were analyzed for CEPsh glia and AWC development phenotypes (Figure 1D). *vab-3(e648)* and *vab-3(ju468)* alleles are nonsense mutations leading to truncations of the homeodomain (Chisholm and Horvitz, 1995; Cinar and Chisholm, 2004). *vab-3(ns157)* and *vab-3(e1796)* alleles have missense mutations in the paired domain and homeodomain, respectively (Zhang and Emmons, 1995; Yoshimura et al., 2008). All *vab-3* mutant alleles examined showed various defects in the expression of the CEPsh identity marker *hlh-17p::GFP* (Figure 1Ci), as shown previously (Yoshimura et al., 2008). In particular, 85-86% of *vab-3(ju468)* and *vab-3(e1796)* mutants lost the expression of *hlh-17p::GFP* in all four CEPsh glia cells (Figure 1Ci).

To determine whether *vab-3* plays a role in general AWC identity, an integrated *odr-1p::TagRFP* marker was crossed into these four *vab-3* mutant alleles. All these *vab-3* mutant alleles displayed completely wild-type expression of the general AWC identity marker *odr-1p::TagRFP* in both AWC neurons (Figure 1B and 1Cii). These results suggest that *vab-3* , unlike *mls-2* , is not required for general AWC identity. These results also suggest that the development of CEPsh glia is not required for general AWC identity.

The AWC axon outgrowth, morphology, and guidance were also examined using the *odr-1p::TagRFP* marker in the *vab-3* mutants. More than 90% of *vab-3(e648)* and *vab-3(ju468)* mutants, while ~20% of *vab-3(e1796)* and *vab-3(ns157)* mutants, exhibited defects in AWC axon development (Figure 1B and 1Ciii), similar to previous results from *vab-3(ns157)* mutants (Yoshimura et al., 2008). To determine whether AWC axon defects correlate with AWC asymmetry defects in *vab-3* mutants and whether CEPsh glia is required for AWC asymmetry, the expression of an integrated AWC ^ON^ asymmetry marker *str-2p::GFP* or *str-2p::TagRFP* was examined in these *vab-3* mutants. Similar to previous results from *vab-3(e648)* mutants (Troemel et al., 1999; Su et al., 2006), the AWC ^ON^ marker was not expressed in either AWC cell in the majority of *vab-3(e648)* and *vab-3(ju468)* mutants (Figure 1B and 1Civ). By contrast, the expression of the AWC ^ON^ marker was normal in *vab-3(e1796)* and *vab-3(ns157)* mutants, (Figure 1Civ). Our results showed that the penetrance of AWC asymmetry defects correlates with that of AWC axon defects. While *vab-3(ju468)* and *vab-3(e1796)* mutants showed a similar degree of CEPsh glia identity defects (Figure 1Ci), they displayed substantially different penetrance of defective AWC axon and AWC asymmetry phenotypes (Figure 1Ciii and 1Civ). These results suggest that *vab-3* has independent functions in the regulation of CEPsh glia development and AWC asymmetry; that *vab-3* regulates AWC asymmetry via controlling AWC axon guidance; and that the homeodomain is essential for VAB-3 function in AWC asymmetry. However, AWC phenotypes of *vab-3(e648)* , which only affects the long isoform *vab-3a* but not short isoforms *vab-3b* or *vab-3c* (Figure 1D), suggest that the homeodomain-only isoforms ( *vab-3b* and *vab-3c* ) are not sufficient for normal AWC development. Together, our results support the notion that CEPsh glia is not required for general AWC identity or AWC asymmetry.

## Reagents

**Table:** 

Strain	Genotype	Source
CB648	*vab-3(e648)* X	(Hodgkin, 1983)
CZ3391	*vab-3(ju468)* X	(Cinar and Chisholm, 2004)
CB3304	*vab-3(e1796)* X	(Zhang and Emmons, 1995)
OS1912	*nsIs105 [hlh-17p::GFP]* I *; vab-3(ns157)* X	(Yoshimura et al., 2008)
OS1914	*nsIs105 [hlh-17p::GFP]* I	(Yoshimura et al., 2008)
IX641	*nsIs105 [hlh-17p::GFP]* I *; oyIs44* [ *odr-1p::DsRed; lin-15(+)* ] V (Lanjuin et al., 2003)	This study
IX6360	*nsIs105 [hlh-17p::GFP]* I *; vyIs56* [ *odr-1p::TagRFP* ] III (Alqadah et al., 2016); *vab-3(ns157)* X	This study
IX2242	*kyIs140 [str-2p::GFP]* I *; vyIs56* [ *odr-1p::TagRFP* ] III (Alqadah et al., 2016)	This study
IX5979	*kyIs140 [str-2p::GFP]* I *; vyIs56* [ *odr-1p::TagRFP* ] III; *vab-3(e648)* X	This study
IX2626	*nsIs105 [hlh-17p::GFP]* I; *vab-3(e648)* X	This study
IX6361	*nsIs105 [hlh-17p::GFP]* I *; vyIs96* [ *str-2p::TagRFP* ] V; *vab-3(ju468)* X	This study
IX5672	*nsIs105 [hlh-17p::GFP]* I *; vyIs96* [ *str-2p::TagRFP* ] V; *vab-3(e1796)* X	This study
IX5989	*nsIs105 [hlh-17p::GFP]* I *; vyIs96* [ *str-2p::TagRFP* ] V; *vab-3(ns157)* X	This study
IX5960	*kyIs140 [str-2p::GFP]* I *; vyIs56* [ *odr-1p::TagRFP* ] III; *vab-3(ju468)* X	This study
IX6357	*kyIs140 [str-2p::GFP]* I *; vyIs56* [ *odr-1p::TagRFP* ] III; *vab-3(e1796)* X	This study
IX6358	*kyIs140 [str-2p::GFP]* I *; vyIs56* [ *odr-1p::TagRFP* ] III; *vab-3(ns157)* X	This study
